# Social Contact Enhances Bodily Self-Awareness

**DOI:** 10.1038/s41598-018-22497-1

**Published:** 2018-03-08

**Authors:** Nesrine Hazem, Morgan Beaurenaut, Nathalie George, Laurence Conty

**Affiliations:** 1 Laboratory of Human and Artificial Cognition (CHArt), Univ Paris Nanterre, Nanterre, France; 2 Institut du Cerveau et de la Moelle épinière (ICM), Social and Affective Neuroscience Laboratory and Centre MEG-EEG, Paris, France; 3 Sorbonne Universités, UPMC Univ Paris 06, UMR_S, 1127 Paris, France; 4 CNRS, UMR 7225 Paris, France; 5 Inserm, U 1127 Paris, France; 6 ENS, Centre MEG-EEG, Paris, France

## Abstract

Human self-awareness is arguably the most important and revealing question of modern sciences. Converging theoretical perspectives link self-awareness and social abilities in human beings. In particular, mutual engagement during social interactions—or *social contact*—would boost self-awareness. Yet, empirical evidence for this effect is scarce. We recently showed that the perception of eye contact induces enhanced bodily self-awareness. Here, we aimed at extending these findings by testing the influence of social contact in auditory and tactile modalities, in order to demonstrate that social contact enhances bodily self-awareness irrespective of sensory modality. In a first experiment, participants were exposed to hearing their own first name (as compared to another unfamiliar name and noise). In a second experiment, human touch (as compared to brush touch and no-touch) was used as the social contact cue. In both experiments, participants demonstrated more accurate rating of their bodily reactions in response to emotional pictures following the social contact condition—a proxy of bodily self-awareness. Further analyses indicated that the effect of social contact was comparable across tactile, auditory and visual modalities. These results provide the first direct empirical evidence in support of the essential social nature of human self-awareness.

## Introduction

In past traditional approaches, cognitive psychologists tended to isolate conscious experience from the social realm. However, cognition is necessarily situated and embedded in a given context, which is, in the case of human beings, mainly a *social* context^1^. This notion of a *situated self* points out the fundamental social dimension of human self-awareness. Social interactions between agents might have been key for the emergence of self-awareness. Supporting evidence for this theory comes from experimental studies on infants^2^ and cross-cultural studies^3^, which showed that the development of social signal systems precedes and informs—as opposed to ‘is derived from’—conceptual representations of self and others. Clinical studies also showed that self-awareness impairments are often associated with difficulties in social signal processing (for example, in autism, in fronto-temporal dementia, and in traumatic brain injury^4^).

In this theoretical framework, it is posited that mutual engagement during social interaction—or *social contact*—provides the infant with awareness of others as ‘attending’ beings, and of him-/her-self as a potential object of that attending^2^. By experimenting the self as the object of another’s attention, infants may develop an initial representation of self and others as psychological entities. Rather than suppressing self-experience in adulthood, social contact would then give rise to the experience of being a cognitive-affective agent^5^. Thus, a fundamental property of social contact throughout the life span would be to enhance self-awareness. However, very few empirical studies have investigated the link between social contact and self-awareness.

Recently, we reported that the perception of a self-directed gaze (that is, of social contact through eye gaze) has the potential to increase self-awareness in adults^6,7^. Gaze is however special in many respects^8,9^. Humans have likely developed an innate sensitivity to eye contact, which is crucial for the development of social cognition^10^ and may predict a selective effect of eye contact on self-awareness. Moreover, real life interactions draw on multiple sensory modalities besides the—most studied—visual modality, including audition and touch, which constitute as important channels for social signaling as eye gaze. Social contact may thus be established by gazing at, touching, or calling out to an interlocutor. Touch is a primary sensory modality, the first of our senses to develop, and our first means of contact with the world^11^. Physical social contact plays a major role in communication and likely has a key contribution to our embodied psychological self^12^. The auditory modality also provides salient social signals^13^. Hearing one’s own first name in particular, by its intrinsic personal meaning, is thought to induce enhanced self-awareness^14,15^, but this has never been formally tested. In order to lay down the social dimension of self-awareness, it is key to demonstrate the influence of social contact on self-awareness across different sensory modalities. This was the aim of the present study.

Self-awareness has multiple dimensions and there are inconsistencies in neuroscientific literature on how to define and quantify them^16,17^. Recent models defend the view that bodily self-awareness or interoceptive awareness (that is, the awareness of the afferent information arising from within the body^18^), is a core element of self-awareness. The remapping of bodily afferent signals in the cortex would underlie our sense of self^19^ and it would be the foundation of the feeling that we exist^20^. Bodily self-awareness can be measured by explicit tasks requiring the participants to count heartbeats for example^21^. However, these tasks have been criticized^22^ and they may fail to capture an essential dimension of bodily self-awareness: Monitoring bodily states would serve the attribution of value to external objects and the making of appropriate decisions towards these objects^20^. Therefore, we chose to confront participants with objects from the outside world and to ask them to monitor their interoceptive reactions to these objects, as an ecological way of assessing bodily self-awareness.

**Figure 1:**
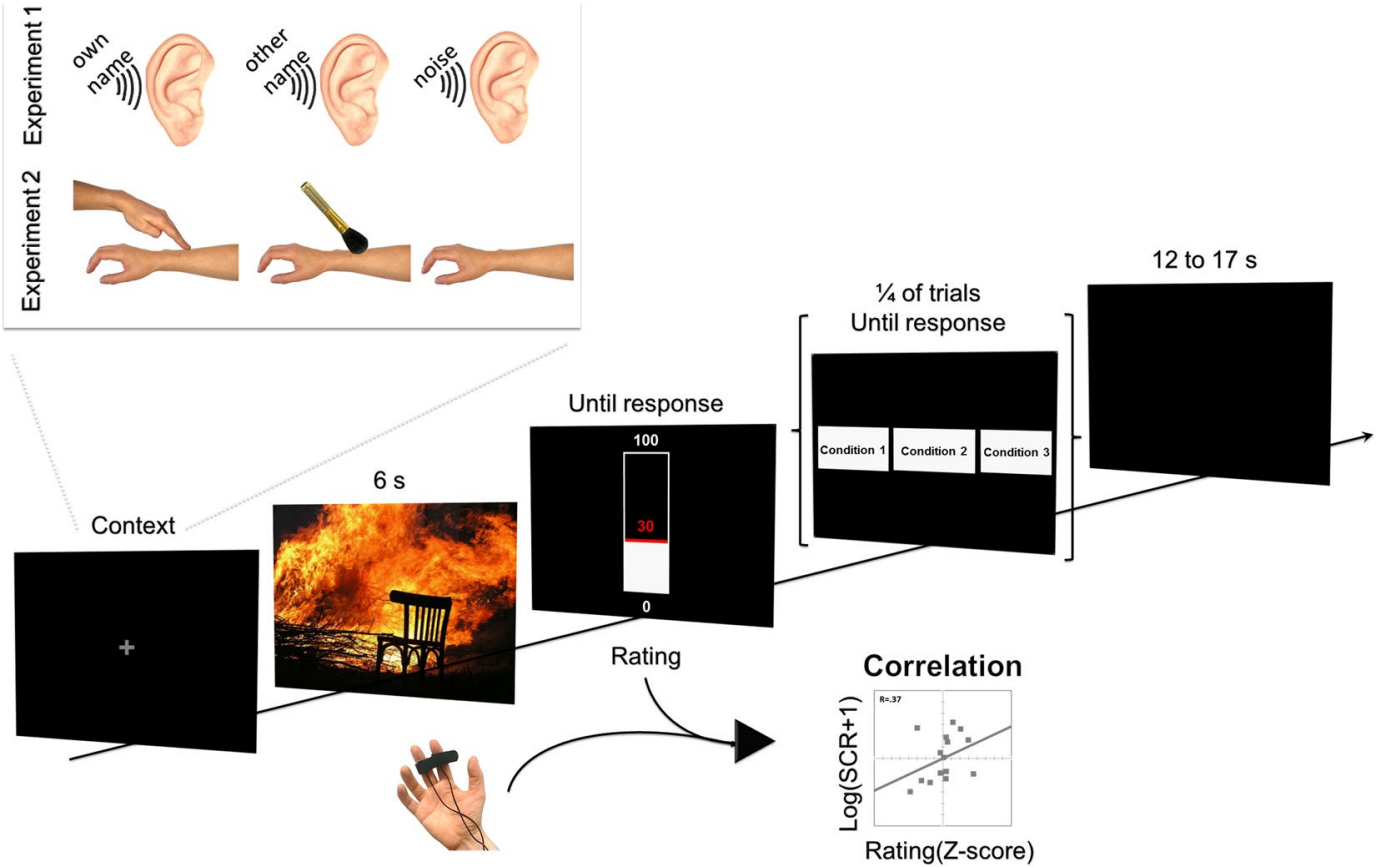
Experimental design: time course of an experimental trial. The emotional picture depicted in the figure is in public domain (from https://pixabay.com/) and is used here for illustrating purpose only. We performed two experiments that varied according to the tested sensory modality of social contact (auditory in the first experiment and tactile in the second experiment). In each experiment, we compared a condition where trials were primed by social contact with two control conditions. In the first experiment, participants were exposed to the sound of their own first name, another unfamiliar name, or white noise, before seeing an emotional picture in response to which they had to rate the intensity of their bodily reaction. In the second experiment, participants were exposed to a human touch, a brush touch, or no-touch, before seeing the emotional picture. The participants’ SCR to the emotional pictures was recorded concomitantly and we computed the correlation between the SCR amplitude and the participant’s rating on a trial-by-trial basis, in each experimental condition for each experiment. For a quarter of the trials, there was an additional, secondary memory task at the end of the trial: a screen display was presented after the participant’s response to the main task, requiring the participant to indicate which context stimulus (Experiment 1: Own Name, Other Name, or Noise; Experiment 2: Human Touch, Brush Touch, or No Touch) had been presented at the beginning of the trial.

We used a paradigm previously developed by our team^6,23^. Participants were asked to rate the intensity of their bodily reactions in response to emotional pictures. We measured concomitantly their Skin Conductance Response (SCR) to the pictures, which reflects general bodily arousal. Indeed, the SCR is well established as an objective measure of bodily arousal in normal subjects^24^ and reflects sympathetic autonomic nervous system activation. Accordingly, it was widely used as an indicator of felt arousal^25,26^. Here, we computed the correlation between the participants’ ratings of their bodily reaction intensity and their SCR amplitude as a proxy of bodily self-awareness. The greater the correlation between subjective ratings and objective physiological measures of bodily reaction to the pictures, the more the participants may be considered aware of their bodily states^16^. Using this paradigm, we already showed that the perception of direct gaze—as compared to that of averted gaze or of a mere fixation cross—increased the bodily self-awareness^6^. Here, we used the same paradigm to test if being called by one’s own first name (Experiment 1) or touched by another individual (Experiment 2) elicit a similar effect (see Fig. 1).

## Results

### Experiment 1: Hearing one’s own name increases bodily self-awareness

**Figure 2:**
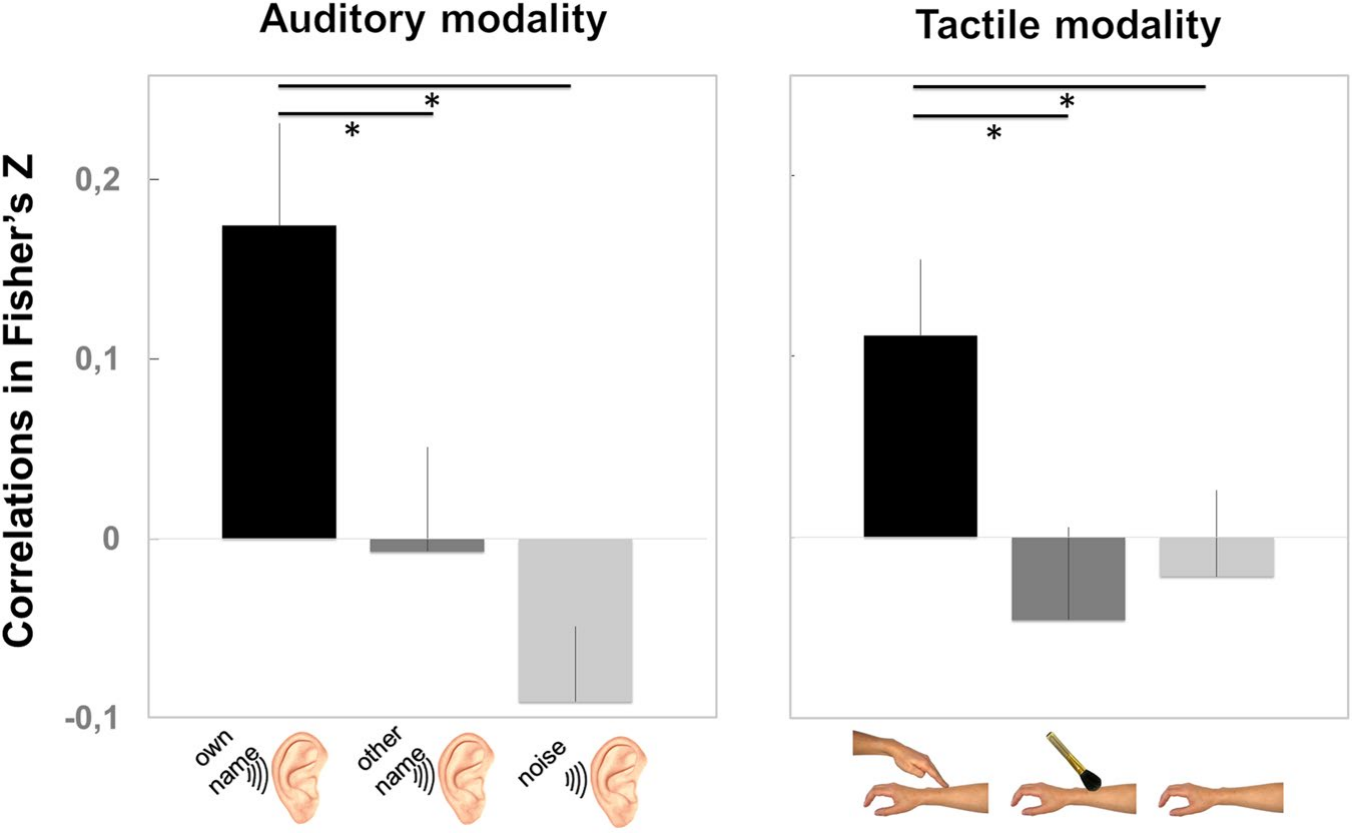
Correlations between skin conductance response (SCR) amplitude and the participants’ rating of the intensity of bodily reactions to emotional pictures. The mean correlation between the participants’ SCR amplitude and their rating is indicated in Fisher’s z scores for each context condition. Left plot: Experiment 1 (N = 28 participants), on the auditory sensory modality, with Own Name/Other Name/Noise conditions. Right plot: Experiment 2 (N = 25 participants), on the tactile sensory modality, with Human Touch/Brush Touch/No-touch conditions. Significant differences are indicated with asterisks (*p < 0.05). Vertical bars represent standard errors of the means.

Consistently with our hypothesis, we found that the correlation between the participants’ ratings and their SCRs amplitude was modulated by the context (*F*_*2,54*_ = 6.90, e_GG_ = 0.96, *P*_*corr*_ = 0.003; ƞ^2^ = 0.20; see Fig. 2). Hearing one’s own name induced a greater value of SCR-rating correlation (*Mean* = 0.17; *SD* = 0.30; 95% Confidence Interval, *CI* = [0.06; 0.29]) than hearing another name (*Mean* = −0.01; *SD* = 0.31; *CI* = [−0.13; 0.11]; *t*_27_ = 2.26, *P* = 0.032) or hearing white noise (*Mean* = −0.09; *SD* = 0.22; *CI* = [−0.18; −0.01]; *t*_27_ = 3.80, *P* = 0.0008). Hearing another name or noise did not lead to significantly different SCR-rating correlation values (*t*_27_ = 1.23, *P* = 0.230). Furthermore, the mean SCR-rating correlation was greater than zero only when participants heard their own name (*t*_27_ = 3.06, *P* = 0.005). Thus, social contact through calling one’s own name enhances bodily self-awareness as assessed by SCR-rating correlation.

### Experiment 2: Being touched by another increases bodily self-awareness

In the next experiment, we examined the effect of social contact through the tactile modality (Fig. 1). As in Experiment 1, we found that participant’s mean SCR-rating correlation was modulated by the context (*F*_*2,48*_ = 4.41, e_GG_ = 0.996, *P*_*corr*_ = 0.0175; ƞ^2^ = 0.16). Being touched by a human induced greater SCR-rating correlation (*Mean* = 0.11; *SD* = 0.21; *CI* = [0.03; 0.20]) than being touched by a brush (*Mean* = −0.05; *SD* = 0.26; *CI* = [−0.15; 0.06]; *t*_24_ = 2.70, *P* = 0.013) or not being touched (*Mean* = −0.02; *SD* = 0.24; *CI* = [−0.12; 0.08]; *t*_24_ = 2.40, *P* = 0.024). Being touched by a brush or not being touched led to similar SCR-rating correlation values (|*t*| < 1). Furthermore, the mean SCR-rating correlation was different from zero only when the participants were touched by a human (*t*_24_ = 2.65, *P* = 0.014). This second experiment demonstrated that social contact established by human touch enhanced the bodily self-awareness of the participants.

Because the perception of a human touch may depend on the interaction between the gender of the person being touched and the gender of the person performing the touch^27^, we ran a supplementary analysis with gender as between-subject factor. There was neither any effect of the participants’ gender nor any interaction between gender and context on the SCR-rating correlations in our Touch experiment (both *Fs* < 1).

Altogether, these findings demonstrate that social contact enhances bodily self-awareness, as measured by the correlations between the participants’ ratings and their SCR amplitude, in both auditory and tactile modalities (see Fig. 2).

### Experiments 1 and 2 - Control analyses

**Figure 3:**
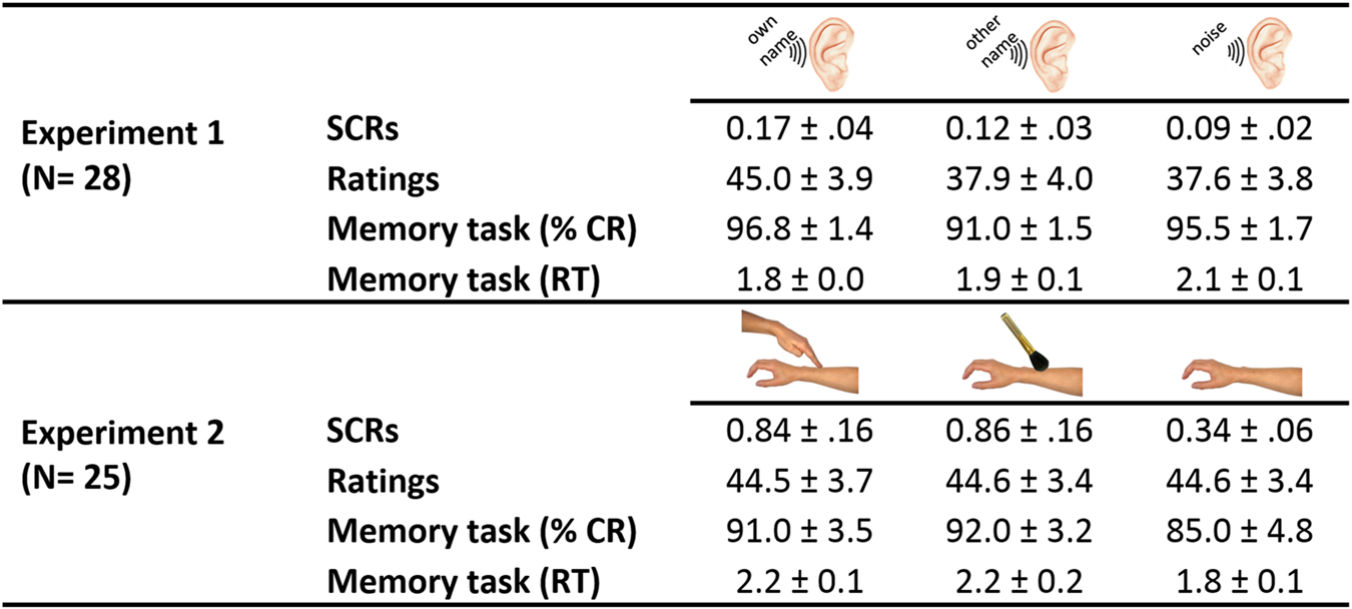
Effect of context on behavioural responses and physiological activity in Experiments 1 (auditory modality) and 2 (tactile modality). All values are expressed as mean ± standard error of the mean. Skin conductance responses (SCRs) are expressed in log(µS + 1). %CR: Percentage of Correct Responses; RT: Reaction Time in seconds.

To deepen our understanding of the effects of social contact, we ran several control analyses (see Fig. 3). Since social contact with another individual has been associated with an increase in arousal^28^, we tested if hearing one’s own name or being touched by another human induced specific physiological or emotional responses *per se* during the emotional picture rating task. We found that in both experiments, the social contact modulated the amplitude of the SCR of the participants (*F*_*2,54*_ = 12.43, e_GG_ = 0.76, *P*_*corr*_ = 0.0002; ƞ^2^ = 0.32 in Experiment 1; *F*_*2,48*_ = 16.06, e_GG_ = 0.68, *P*_*corr*_ = 0.0001; ƞ^2^ = 0.40 in Experiment 2). Hearing one’s own name led to higher SCR than hearing another name (*t*_27_ = 3.60, *P* = 0.001) and hearing white noise (*t*_27_ = 4.00, *P* = 0.0005). Hearing another name also increased SCR when compared to hearing noise (*t*_27_ = 2.35, *P* = 0.027). Being touched either by another human or by a brush elicited higher SCR than not being touched (*t*_24_ = 4.13, *P* = 0.0004 and *t*_24_ = 4.32, *P* = 0.0002, respectively). However, being touched by a human did not elicit higher SCR than being touched by a brush (|*t*| < 1). In addition, hearing one’s own name modulated the participants’ ratings of the intensity of their bodily reactions (*F*_*2,54*_ = 17.36, e_GG_ = 0.97, *P*_*corr*_ = 0.000002; ƞ^2^ = 0.39). The own name condition led to higher ratings when compared to the other name (*t*_27_ = 4.69, *P* = 0.00007) and noise (*t*_27_ = 5.14, *P* = 0.00002) conditions; the latter did not differ from each other (|*t*| < 1). By contrast, being touched by a human did not modulated the participant’s ratings (|*t*| < 1).

In order to investigate the possible effect of the context condition on participant’s attention to the context stimulus, we also analysed the secondary memory task that was performed in a quarter of the trials (see Fig. 1). The mean percentage of correct responses (%CR) in this task was excellent in both experiments (*M*ean = 94.4%, *SD* = 8.2), ensuring that the participants paid attention to the context stimuli. The overall mean response time (RT) was 1.9 s (*SD* = 0.4 s) (Fig. 3). The analyses performed on %CR and RT did not reveal any significant effect of the social contact on these variables in either experiment (all *Fs* < 1).

### Experiment 1, 2 and Baltazar *et al*.^6^: The effect of social contact on bodily self-awareness is comparable across sensory modalities

**Figure 4:**
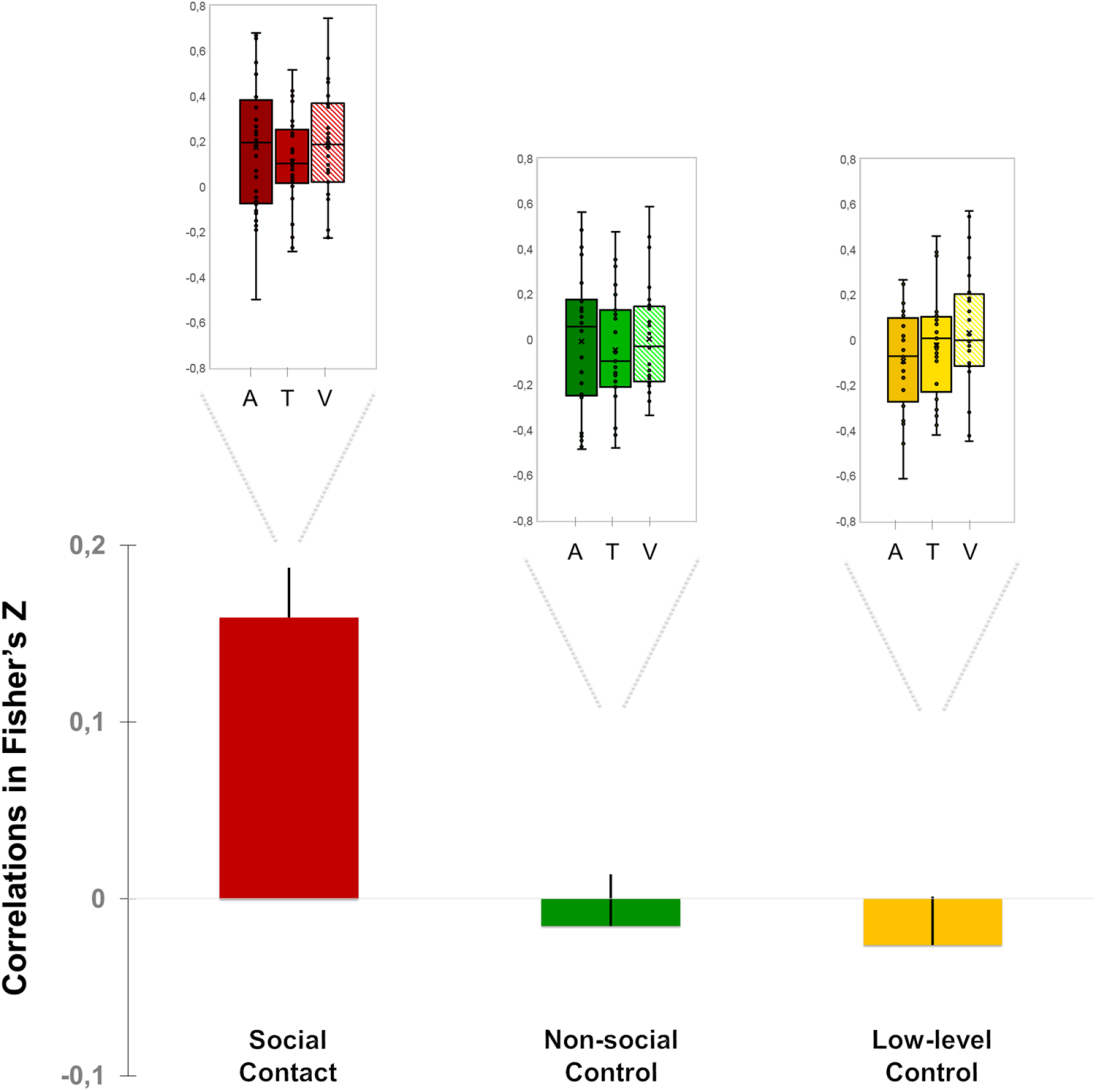
Correlation between participants’ skin conductance responses (SCR) amplitude and subjective ratings as a function of social contact across different sensory modalities. The main bar plot represents the overall mean participants’ correlation (in Fisher’s z scores) between SCR amplitude and subjective rating for the Social Contact (in red), Non-social Control (in green), and Low-level Control (in yellow) conditions, averaged across Experiment 1 on auditory modality (A), Experiment 2 on tactile modality (T), and the experiment of Baltazar *et al*.^6^ on visual modality (V). The vertical lines on the bars represent the standard errors of the means. Above each bar of the main plot, an inset detailing the data of each experiment is presented: the points represent the participants’ correlations in Fisher’s z scores for each context condition (from left to right: Own Name/Human Touch/Eye Contact for the Social Contact conditions, in shades of red; Other Name/Brush Touch/Averted Gaze for the Non-social Control conditions, in shades of green; White noise/No-touch/Cross conditions for the Low-level Control conditions, in shades of yellow). For each condition, the box plots show the lower (Q2) and upper (Q3) quartiles, and the horizontal bar inside the box represent the median value of SCR-rating correlation (in Fisher’s z score). Vertical bars outside the box represent the distribution range, with the upper bound corresponding to the maximal individual correlation and the lower bound corresponding to the lowest individual correlation.

Finally, to test the extent to which the effect of social contact on bodily self-awareness depended on the sensory modality of the social contact, we ran an analysis including the data of experiments 1 and 2 and the data of Baltazar *et al*.^6^. We found that the participants’ accuracy in judging the intensity of their bodily emotionaly arousal state was similar across the three experiments (*F*_*2*,78_ = 1.02, *P* = 0.37; ƞ^2^ = 0.03). Importantly, social contact increased the correlation between the participants’ ratings and their SCR amplitude (*F*_*2,156*_ = 14.85, e_GG_ = 0.99, *P*_*corr*_ < 0.000001; ƞ^2^ = 0.16) with similar magnitude across auditory, tactile and visual modalities (*F*_*4,156*_ = 0.74, e_GG_ = 0.99, P_*corr*_ = 0.57; ƞ2 = 0.02). Overall, irrespective of sensory modality, social contact induced a greater value of SCR-rating correlation (*Mean* = 0.16; *SD* = 0.25; *CI* = [0.10; 0.22]) than each control condition (for the Non-social Control condition: *Mean* = −0.02; *SD* = 0.27; *CI* = [−0.07; 0.04], *t*_78_ = 4.43, *P* = 0.00003; for the Low-level Control condition: *Mean* = −0.03; *SD* = 0.25; *CI* = [−0.08; 0.03], *t*_*78*_ = 4.89, *P* < 0.000001 – see Fig. 4). In contrast, the control conditions were not different from each other (|*t*| < 1).

## Discussion

Following a previously established procedure^6,23^, we showed that, irrespective of its sensory modality, social contact enhances bodily self-awareness. While cognitive psychologists have long tended to consider mental functions—including self-awareness—as isolated entities, recent arguments pointed out the fundamental social nature of self-awareness^29^. Our results provide a strong empirical support for this theoretical view.

What are the cognitive mechanisms that may mediate the effect of social contact on bodily self-awareness? In an effort to answer this question in relation to the effect of eye contact on self-awareness, it was recently proposed that direct gaze has a self-referential power^7^. It would automatically trigger a cognitive background centred on the self, manifesting itself through four types of eye contact effects: the enhancement of self-awareness, memory effects, the activation of pro-social behaviour, and positive appraisals of others. Interestingly, social contact through tactile modality induces similar effects. Social touch promotes pro-social behaviours^30^, modulates the perception of others by driving more positive assessments^31^, and stimulates public self-awareness as measured using a Likert scale^32^. Moreover, social contact through auditory modality shares common mechanisms with eye contact. Hearing one’s own name activates similar brain structures as the perception of eye contact, in adults^33^. “Baby-talk” (i.e. speech directed toward the child) of a mother motivates ocular exploration of the environment in 6-months babies just as eye contact does^34^. Together with these findings, our results suggest that whatever the sensory modality, social contact has a self-referential power. This view ties nicely with neuroimaging studies that demonstrated common brain networks for social signal processing and self-related processing (i.e. the Default Mode Network^1^). One may speculate that social contact reinforces the functional connectivity between structures of the Default Mode Network, thus boosting self-related process. However, future studies are needed to elucidate the neural basis of the effect of social contact on self-awareness.

It may also be suggested that the increase in bodily self-awareness was mediated by different mechanisms according to the sensory modality^16,35^. For the auditory modality, we reported a greater arousal in the own name than the control conditions. This may be explained by the high intrinsic saliency of the own name. Its foremost personal relevance makes it a highly salient stimulus known to automatically capture attention, even in the absence of conscious perception^36^. Yet, it is unlikely that the more arousing nature of the Own Name condition accounted for the participants’ greater bodily awareness in this condition. Admittedly, stimuli that elicit higher arousal may induce more salient bodily signals that are easier to evaluate^37^. However, we did not observe a significant difference on SCR-ratings correlation between the Other Name and Noise conditions, while these conditions also differed significantly on SCR amplitudes. Moreover, the effects of social touch (experiment 2) and of eye contact^6^ on bodily self-awareness were not concomitant with any main effect of arousal, while the effect of social contact on participant’s bodily self-awareness was not significantly different across sensory modalities (auditory, tactile, and visual). Taken together, the greater interoceptive awareness associated with social contact is likely not related to an increase of arousal, but rather hinged upon a mechanism allowing greater access to bodily states.

There is discrepancy in the literature on how to measure bodily self-awareness^17^. It has been recommended to compare participant’s self-reports with physiological data^16^. Accordingly, most neuroscientific studies measured bodily self-awareness - or “interoceptive awareness” - using comparison between subjective-reports and physiological signals in the form for example of electrodermal activity^38^. In this line, we developed a paradigm to measure bodily self-awareness by the correlation between subjective-reports and skin conductance responses^6,23^. In our experiments, participants were required to rate the intensity of their internal bodily reactions in response to emotional pictures. Importantly, there were not required to focus on a particular physiological cue (e.g. heart rate, skin sweating, or respiration). We thus collected the ratings of their general bodily arousal state. As mentioned in introduction, the SCR is a well established, objective measure of bodily arousal, reflecting sympathetic autonomic nervous system activation. Thus, the correlation between participant’s report of their internal bodily arousal state and the amplitude of their SCR reflected their accuracy in judging their bodily arousal state, which we took as a proxy of bodily awareness.

In Experiment 2, we used a specific type of human touch, known to activate the C-Tactile (CT) afferents nerve fibers, a class of unmyelinated mechanosensitive fibers that innervate the human hairy skin. The CT afferents have been described as the sensory pathway of social touch as they respond selectively to gentle, slow skin stroking or caress-like tactile stimulations, in contrast to the tactile fibers A and B that subserve discriminative touch^39^. Importantly however, the tactile stimulations delivered in the brush touches also presented the characteristics necessary to activate CT afferents. Therefore, the greater bodily self-awareness observed in the human touch condition cannot rely merely on a stimulus-driven activation of the CT neural pathway. This reinforces the view that the effects of own name, social touch, and eye contact on bodily awareness were subtended by common brain mechanisms. The results of experiment 2 also suggest that knowing that the contact is directly created by another human agent is essential to the effect of social contact on bodily self-awareness. This fits with the result that we recently obtained in relation to eye contact. Using the same paradigm as in the present study, we showed that the belief of being watched was necessary to elicit an increase of bodily self-awareness in a situation where participants faced another individual wearing opaque sunglasses^23^. Our work converges with the recent accounts of interoception that defend a key role of priors in interoceptive processing, concomitantly with bottom-up signals—i.e. ascending bodily viscerosensory signals^40^.

There is evidence that during phylogenesis, humans have developed an innate sensitivity to eye contact^10,41^. In parallel, the sensitivity to first name and social touch appears very early during development. The own name is a socially constructed cue since it is given by others. However, it acquires meaning early on by repetitive hearing. From 4- to 5-months of age, infants prefer to listen to their own rather than other names^42^, and they use it as a social cue to guide their attention to events and objects in the external word^43^. Moreover, touch is the first sense to emerge in utero and the most strongly developed at birth. It is one of the earliest and most fundamental forms of parent-child communication^11^. Early tactile deprivation has been shown to affect socio-cognitive development with long-lasting effects, even in the case of later normal care-giving^44^. On the other hand, only few minutes of additional tactile stimulation per day allow the exhibition of a range of positive developmental effects^45^. These studies point out the early sensitivity to several types of social contact signals and its importance in infant development. This supports the view that the self-referential property of social contact plays an essential role in the emergence of infant’s self-awareness.

In conclusion, we showed that hearing one’s own name spoken by another person and being touched by another person both increased bodily self-awareness, just as perceiving direct gaze does^6^. This provides the first empirical demonstration that social contact irrespective of sensory modality elicits bodily self-awareness. In doing so, it supports the notion of the social nature of the self—i.e. that human self-awareness emerges in interpersonal contacts.

## Methods

### Subjects

Thirty-five adults (mean age = 22 years, SD = 2.6; 19 men) participated in experiment 1 and thirty-two different adults (mean age = 24 years, SD = 3.4; 15 men) participated in experiment 2. We based our sample size calculation on the previous results obtained in our group regarding the effect of social contact through visual modality on bodily self-awareness^6,23^. The minimum effect size f in these studies was 0.42. Based on this value, we computed a sample size of 24 for a power of 0.8, at alpha 0.05 (with a medium value of non sphericity of 0.75) using the software G*Power 3^46^. We then included thirty-five participants in Experiment 1 and thirty-two in Experiment 2 with the aim of accounting for potential exclusion of participants due to technical errors or outlier values.

For both experiments, all participants had normal or corrected-to-normal vision and no history of neurological or psychiatric illness. All participants were right-handed, French speakers, and naïve to the goal of the experiments. For experiment 1, all participants had normal audition and for experiment 2, the participants did not have any chronic skin disease. Informed written consent was obtained from every participant in accordance with the 1964 Declaration of Helsinki and its later amendments. All procedures were approved by the local ethics committee (CPP Ile de France III, approval n°C07-28). All the methods used in our experiments were performed in accordance with the guidelines and regulations of the local ethics committee. Each participant received a payment of 20 Euros for his/her participation. Four participants in experiment 1 were excluded due either to technical error (N = 3), or because the ‘Other Name’ was not unfamiliar (N = 1). In experiment 2, two participants were excluded due to health issue (eczema on the forearm for one participant, and temporary fainting during the experiment for the other participant). After additional outlier exclusion (see below), the final data sample included 28 participants (mean age = 22 years, SD = 2.4; 14 men) for the first experiment, and 25 participants (mean age = 24 years, SD = 4.6; 11 men) for the second experiment.

### Stimuli

#### Emotional stimuli

The emotional stimuli displayed in both experiments were the same forty-eight emotional pictures (24 positive and 24 negative) as in Baltazar *et al*.^6^ and Hazem *et al*.^23^. They were selected from the International Affective Picture Systems^47^ to induce emotional experience. The full procedure of stimulus selection is described in Baltazar *et al*.^6^.

#### Context stimuli, Experiment 1

Thirty-two audio recordings of first names (female voice in half of the cases, male in the other half) were created to generate the ‘Own Name’ and ‘Other Name’ contexts. The uttered name was the name of the participant in half of the audio recordings (‘Own Name’ context) and another unfamiliar name in the other half (‘Other Name’ context), for both the male and the female voices. They were uttered with a calling intonation to ensure a communicative intent. The’Own Name’ and the ‘Other Name’ were matched in terms of gender, duration (ranging from 500 to 600 ms), frequency (medium frequency of occurrence of 15,000 to 30,000 times in France between 1980 and 2000: see https://dataaddict.fr/prenoms/) and phonetic properties (same number of syllables, with no more than three syllables). The loudness of the ‘Own Name’ and the ‘Other Name’ was normalized with the AUDACITY 2.0.6 freeware. Moreover, prior to the experiment, each subject provided the first names of their five closest friends and of their relatives (parents, siblings, romantic partner, and children), in order not to select any of these names for the ‘Other Name’ condition. At the end of the experiment, we asked the participant whether the ‘Other Name’ was familiar to him/her and we eliminated from the analysis the only participant for whom it was the case. The white noise for the Low-level control condition was generated with AUDACITY 2.0.6, with a duration of 450, 500, 550, 600, or 650 ms.

#### Context stimuli, Experiment 2

Tactile stimulations in both Human and Brush touch conditions were gentle strokes delivered with a pressure of approximately 8 Pa and a velocity of about 4 cm/s, aimed at inducing optimal activation of the CT afferents neural pathway, thought to be the channel of social touch^48^. All stimulations were proximo-distal in orientation. In the Human Touch condition, the stimulations were performed by the male experimenter MB using his joined index and middle fingers. In the Brush Touch condition, the stimulations were performed with a makeup goat hair brush (3 cm diameter). Both types of stimulation were delivered on 2 different skins regions of 8 cm length on the participant’s left forearm, previously delineated with a washable marker. The stimulations were alternating between the two skin regions to minimise the rapid habituation of CT fibers, which require a 30 seconds inter-trial period to return to their baseline level^49^.

Before the experiment, the stimulations were practiced by the experimenter and video-recorded to verify stroking velocity and pressure. During the experiment, the experimenter was visually guided by a cursor moving on a screen in order to achieve the correct speed and length of the touch. Moreover, the stroking velocity was evaluated post-experiment from the video-recordings. For this, we randomly extracted 100 video recordings of the touches performed during the experiment (50 human touch and 50 brush touch videos). The speed of the touches was computed and we performed a repeated-measures ANOVA on the measured speed with Context (Human Touch/Brush Touch) as within-subject factor. This showed that there was no significant difference between the Human and the Brush Touch conditions (*Mean* = 4.22 ± 0.10 cm/s; *F*_*1,98*_ = 1.63, *P* = 0.20).

### Experimental design

The participants were seated at a distance of 70 cm from a 22-inch computer screen with a resolution of 1280 × 1024 pixels. Each trial was initiated by the presentation of a fixation cross of 3-by-3 degrees of visual angle. After a random duration of 0.8 to 1.5 s, it was followed by the presentation of the context stimulus.

#### Experiment 1

The auditory context stimulus was delivered binaurally through headphones, at about 75 dB SPL maximal intensity. This context stimulus was the name of the participant (Own Name condition), another unfamiliar name (Other Name condition)—uttered by a female (in half of the cases) or a male voice (in the other half), or a white noise (Noise condition). Each condition was presented in one-third of the trials.

#### Experiment 2

The context stimulus was the slow gentle touch done with the hand of the experimenter (Human Touch condition), the slow gentle touch done by the experimenter using a brush (Brush Touch condition), or no-touch (No-touch condition). The participant’s touched arm was placed behind a curtain so that the experimenter and the type of touch performed were not visible.

***In both experiments***, we used 16 trials per context condition (for a total of 48 trials). The order of the conditions was pseudo-randomized so that any given experimental condition was not repeated more than two times in a row. In each trial, the context stimulus was followed by the emotional picture, so that the delay between the context stimulus onset and the emotional picture onset was 1.5 s in Experiment 1 and 2.5 s in Experiment 2. The emotional picture was presented for 6 s. It covered 39-by-24 degrees of visual angle, in the centre of the screen. It was immediately followed by a continuous vertical scale ranging from 0 to 100, which was displayed until the participant provided his/her response. The participants were instructed to focus on the internal bodily changes that the emotional picture caused in them during its presentation and to evaluate on this basis the intensity of their emotional reaction from 0 (not at all intense) to 100 (very intense) by moving a vertical cursor along the scale. It was specified that the bottom of the scale corresponded to feeling completely relaxed, quiet, calm, and without any particular bodily change induced by the picture, whereas the top of the scale corresponded to feeling very stimulated, excited or nervous, with intense bodily reaction to the picture. In addition, for a quarter of the trials, a new screen appeared after the participant’s response, displaying the following words, one alongside the other: “Propre Prénom”, “Autre Prénom”, and “Bruit” (*Own Name*, *Other Name*, and *Noise*) for the first experiment, or “Toucher Humain”, “Toucher Pinceau”, and “Pas de Toucher” (*Human Touch*, *Brush Touch*, and *No-touch*) for the second experiment. The participant had to indicate which type of context stimulus had been presented before the emotional picture. This constituted a secondary memory task to ensure that participants paid attention to the context stimuli. Between trials, the screen remained black for a duration that varied randomly between 12 and 17 s in order to allow skin conductance to return to baseline level. The association between the context image and the emotional stimulus was counterbalanced across participants so that every emotional picture (N = 48) was seen once during the experiment with always 8 positive and 8 negative pictures associated with each context condition.

### Physiological recordings and analysis

Physiological responses were recorded using ADInstruments© (ML870/Powerlab 8/30) acquisition system.

#### Skin conductance response (SCR)

Two Ag–AgCl electrodes filled with isotonic NaCl unibase electrolyte were attached to the palmar surface of the middle phalanges of the index and middle fingers of the non-dominant hand. The raw SCR signals were recorded at a sampling rate of 2 kHz, amplified and low-pass filtered online at 10 Hz. Then, the SCR was offline downsampled at 2 Hz. Trials containing artefacts or noisy baseline (i.e. mean of activity during the second preceding the context onset) were manually rejected (less than 8% of the trials). The SCR in response to the emotional picture was defined as the maximum change from the baseline level occurring between 1 and 6 s after the emotional picture onset. The SCR amplitudes were then log-transformed [Log (μS + 1)] to normalize the data^25^.

#### Correlations between SCR and subjective ratings of emotional response intensity

The two experiments were analysed independently from each other. For each experiment, we computed the correlation (Pearson’s r coefficient) between the SCR evoked by the emotional pictures and the ratings provided by the participants, in each context condition separately and for each participant. Correlation r values are typically not normally distributed^50,51^. Therefore, we performed Fisher’s r-to-z transformation in order to normalize the correlation values. Shapiro-Wilk statistical tests confirmed that the Fisher z scores distribution did not significantly differ from a normal distribution, for each condition in both experiments (all P > 0.20). We then screened the Fisher’s z scores for extreme values. Three participants in the first experiment and five participants in the second experiment had outlier values (Fisher’s z scores differing by more than two standard deviations from the group mean in one or more experimental conditions); they were excluded from all subsequent analyses.

### Statistical analyses

#### Statistical analysis of the SCR-rating correlation data

For each experiment, a one-way repeated-measures ANOVA with Context (Experiment 1: Own Name/Other Name/Noise and Experiment 2: Human Touch/Brush Touch/No-touch) as within-subject factor was run on the SCR-rating correlations transformed into Fisher’s z scores. We used the Greenhouse-Geisser correction to correct for deviations from the assumption of sphericity (the ε_GG_ correction factor and the corrected *P* or *P*_*corr*_ are reported). Effect sizes (eta-squared, η^2^) are reported together with F and p values of the main effects. Planned comparisons between the conditions taken two-by-two were further performed using bilateral, paired Student t-tests when main effects were observed.

### Control analyses

#### Main effects of context on arousal and ratings

For each experiment, additional one-way repeated-measures ANOVAs with Context as within-subject factor were carried out on SCRs and on subjective ratings, respectively.

#### Secondary memory task

For each experiment, one-way repeated-measures ANOVAs with Context as within-subject factor were run on the RTs and on the %CR obtained in the secondary memory task.

#### Control analysis specific to the second experiment

A repeated-measures ANOVA with Context (Human Touch/Brush Touch/No-touch) as within-subject factor and participant’s gender as between-subject factor (Female/Male) was run on the Fisher’s z scores of SCR-rating correlations.

#### Analysis across sensory modalities: Experiment 1, 2, and Baltazar *et al*.^6^

A repeated-measures ANOVA with Experiment (Experiment 1/Experiment 2/Experiment from Baltazar *et al*.^6^) as between-subject factor and Condition (Social Contact condition: Own Name – Human Touch - Eye Contact/Non-social control condition: Other Name - Brush Touch - Averted Eyes/Low-level control condition: Noise - No-touch – Fixation cross) as within-subject factor was run on the Fisher’s z scores of SCR-rating correlations.

### Data availability

The datasets generated and/or analysed during the current study are available from the corresponding author on request.

## Electronic supplementary material

Individual correlational r values
