# Supplementary material for: Social Contact Enhances Bodily Self-Awareness
Source: Sci Rep. 2018 Mar 8;8:4195. doi: 10.1038/s41598-018-22497-1 (PMC5843600; doi:10.1038/s41598-018-22497-1)
Supplement: Supplementary file 1 — Individual correlational r values [file 41598_2018_22497_MOESM1_ESM.docx]

# Supplementary information

# social contact enhances BODILY self-awareness

Nesrine HAZEM^1,2,3*^, Morgan BEAURENAUT^1,2^, Nathalie GEORGE^2,3,4,5,6^ **^£^**, Laurence CONTY^1^ **^£^**

^1^ Laboratory of Human and Artificial Cognition (CHArt), Univ Paris Nanterre, Nanterre, France

^2^ Institut du Cerveau et de la Moelle épinière (ICM), Social and Affective Neuroscience Laboratory and Centre MEG-EEG, Paris, France

^3^ Sorbonne Universités, UPMC Univ Paris 06, UMR_S 1127, Paris, France

^4^ CNRS, UMR 7225, Paris, France

^5^ Inserm, U 1127, Paris, France

^6^ ENS, Centre MEG-EEG, Paris, France

**£** Co-last authors

Number of Supplementary Tables: 2

* Corresponding author at: Institut du Cerveau et de la Moelle épinière (ICM), Social and Affective Neuroscience Laboratory, GH Pitié Salpêtrière, 47 bd de l'hôpital, F-75013 Paris. Tel.: +33 1 57 27 43 10. E-mail address:

**Supplementary Table S1. Individual correlational r values between participant’s skin conductance response amplitude and their ratings of the intensity of their bodily reactions to emotional pictures for each condition in Experiment 1 (auditory modality: Own Name / Other Name / Noise).**

| **Experiment 1**  **(N= 28)** | 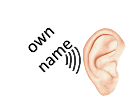 | 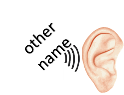 | 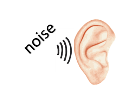 |
| --- | --- | --- | --- |
|  | 0.592 | 0.168 | -0.210 |
|  | -0.017 | -0.419 | -0.044 |
|  | 0.586 | 0.246 | -0.343 |
|  | 0.070 | 0.177 | -0.098 |
|  | 0.244 | 0.388 | 0.263 |
|  | 0.229 | -0.079 | 0.002 |
|  | -0.104 | -0.189 | 0.167 |
|  | -0.116 | -0.440 | -0.035 |
|  | -0.064 | -0.238 | 0.020 |
|  | 0.506 | 0.075 | 0.110 |
|  | -0.045 | 0.138 | -0.354 |
|  | -0.459 | 0.124 | -0.090 |
|  | -0.147 | 0.363 | 0.245 |
|  | 0.261 | -0.244 | -0.073 |
|  | -0.186 | 0.141 | -0.069 |
|  | 0.240 | -0.142 | -0.280 |
|  | 0.206 | 0.142 | 0.164 |
|  | 0.378 | -0.392 | -0.348 |
|  | 0.338 | -0.184 | -0.216 |
|  | 0.185 | -0.181 | -0.428 |
|  | -0.170 | -0.450 | -0.283 |
|  | 0.461 | -0.250 | 0.028 |
|  | 0.136 | 0.039 | -0.134 |
|  | 0.046 | 0.360 | -0.546 |
|  | -0.075 | 0.451 | -0.163 |
|  | 0.501 | 0.511 | 0.131 |
|  | 0.289 | 0.100 | 0.064 |
|  | 0.577 | -0.402 | 0.127 |

**Supplementary Table S2. Individual correlational r values between participant’s skin conductance response amplitude and their ratings of the intensity of their bodily reactions to emotional pictures for each condition in Experiment 2 (tactile modality: Human Touch / Brush Touch / No-touch).**

| **Experiment 2**  **(N= 25)** | 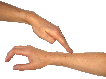 | 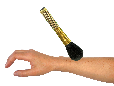 | 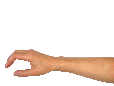 |
| --- | --- | --- | --- |
|  | 0.361 | -0.397 | -0.074 |
|  | 0.402 | 0.198 | 0.036 |
|  | -0.050 | -0.122 | -0.321 |
|  | 0.041 | -0.444 | 0.095 |
|  | 0.093 | 0.341 | -0.092 |
|  | 0.076 | -0.204 | -0.395 |
|  | 0.151 | 0.131 | -0.191 |
|  | 0.125 | -0.094 | 0.108 |
|  | 0.382 | 0.313 | 0.431 |
|  | 0.105 | -0.155 | -0.055 |
|  | -0.278 | 0.045 | 0.125 |
|  | 0.234 | -0.245 | -0.356 |
|  | 0.063 | -0.183 | -0.359 |
|  | 0.028 | 0.240 | 0.016 |
|  | 0.166 | -0.144 | 0.010 |
|  | 0.477 | 0.113 | 0.358 |
|  | -0.164 | -0.172 | 0.072 |
|  | 0.004 | -0.057 | 0.373 |
|  | -0.263 | -0.388 | 0.091 |
|  | 0.052 | -0.372 | -0.254 |
|  | 0.118 | 0.443 | 0.102 |
|  | 0.263 | 0.095 | 0.112 |
|  | 0.283 | -0.205 | -0.019 |
|  | -0.219 | 0.035 | -0.297 |
|  | 0.222 | 0.132 | -0.036 |
